# In Vitro Prevascularization of Self-Assembled Human Bone-Like Tissues and Preclinical Assessment Using a Rat Calvarial Bone Defect Model

**DOI:** 10.3390/ma14082023

**Published:** 2021-04-17

**Authors:** Fabien Kawecki, Todd Galbraith, William P. Clafshenkel, Michel Fortin, François A. Auger, Julie Fradette

**Affiliations:** 1 Centre de Recherche en Organogénèse Expérimentale de l′Université Laval/LOEX, Division of Regenerative Medicine, CHU de Québec Research Center-Université Laval, Québec, QC G1J 1Z4, Canada; fabien.kawecki.1@ulaval.ca (F.K.); Todd.Galbraith@crchudequebec.ulaval.ca (T.G.); bill.clafshenkel@gmail.com (W.P.C.); Michel.Fortin@fmd.ulaval.ca (M.F.); Francois.Auger@fmed.ulaval.ca (F.A.A.); 2 Department of Surgery, Faculty of Medicine, Université Laval, Québec, QC G1V 0A6, Canada; 3 Faculty of Dentistry, Université Laval, Québec, QC G1V 0A6, Canada; 4 Service of Oral and Maxillofacial Surgery, CHU de Québec-Université Laval, Québec, QC G1J 1Z4, Canada

**Keywords:** prevascularization, mesenchymal stem cells, calvarial bone defect, self-assembly, endothelial, adipose

## Abstract

In vitro prevascularization has the potential to address the challenge of maintaining cell viability at the core of engineered constructs, such as bone substitutes, and to improve the survival of tissue grafts by allowing quicker anastomosis to the host microvasculature. The self-assembly approach of tissue engineering allows the production of biomimetic bone-like tissue constructs including extracellular matrix and living human adipose-derived stromal/stem cells (hASCs) induced towards osteogenic differentiation. We hypothesized that the addition of endothelial cells could improve osteogenesis and biomineralization during the production of self-assembled human bone-like tissues using hASCs. Additionally, we postulated that these prevascularized constructs would consequently improve graft survival and bone repair of rat calvarial bone defects. This study shows that a dense capillary network spontaneously formed in vitro during tissue biofabrication after two weeks of maturation. Despite reductions in osteocalcin levels and hydroxyapatite formation in vitro in prevascularized bone-like tissues (35 days of culture), in vivo imaging of prevascularized constructs showed an improvement in cell survival without impeding bone healing after 12 weeks of implantation in a calvarial bone defect model (immunocompromised male rats), compared to their stromal counterparts. Globally, these findings establish our ability to engineer prevascularized bone-like tissues with improved functional properties.

## 1. Introduction

The “gold standard” procedure for maxillofacial reconstruction remains autologous bone grafting [1,2]. However, this option is not without several drawbacks, including donor site morbidity [3,4,5]. Therefore, other options have been developed, such as the use of allogeneic matrices [6,7], xenogeneic materials [8,9], or synthetic bone substitutes [10,11,12,13,14]. However, these substitutes are often acellular, can elicit immunogenic responses, and possess limited osteointegration potential (reviewed in [15,16,17]). Developments in the field of bone tissue engineering propose to combine biomaterial scaffolds, human cells, and/or growth factors to produce tridimensional (3D) living and functional biomimetic bone substitutes for clinical applications [18,19,20,21,22,23]. Nonetheless, challenges remain, such as the need to engineer bone substitutes that replicate the volume and the structure of native bone surrounding the defects [24]. These defects may require voluminous tissues that exceed the limit of oxygen and nutrient diffusion (>200 µm); thus, cell survival may be compromised at the core of the tissue-engineered bone [25,26]. To improve survival in these constructs, in vitro prevascularization approaches have been developed to obtain a microvasculature system that can connect with the host’s vasculature via anastomoses upon implantation [27,28,29,30,31,32].

The combination of the self-assembly approach of tissue engineering with the appropriate stem cells induced towards osteogenic differentiation allows for the production of autogenous bio-inspired bone-like tissues without harvesting bone from a donor site [33,34]. Indeed, our research team has produced a human self-assembled model of bone-like tissue composed of mineralized extracellular matrix (ECM) secreted by osteogenically-differentiated human adipose-derived stromal/stem cells (hASCs) [33]. This approach harnesses the intrinsic capacity of ASCs to secrete and assemble endogenous ECM components upon long-term ascorbic acid supplementation, generating a natural human scaffolding. In the present study, we hypothesize that the addition of an endothelial cell-based capillary network could favor osteogenic differentiation and in vitro mineralization during the production of the tissues. The ability of these prevascularized constructs to subsequently improve bone repair, as well as graft survival, was analyzed in a model of rat calvarial bone defects.

Our results show that capillary networks were spontaneously formed in vitro within the human tissues composed of osteogenically-differentiated hASCs. These tissues showed a more organized capillary network compared to prevascularized stromal tissues containing non-osteogenically-differentiated hASCs. Our results also revealed that prevascularization of the bone-like tissues with human umbilical vein endothelial cells (HUVECs) was associated with significantly reduced osteocalcin (OCN) levels, a marker associated with the later phases of in vitro osteogenesis and biomineralization. In addition, in vivo data suggested that implantation of these prevascularized bone-like tissues improves survival in a rat model of calvarial bone defects after 12 weeks, without untoward effects on bone healing, when compared to prevascularized stromal tissues and bone-like tissues engineered without prevascularization.

## 2. Materials and Methods

### 2.1. Human Adipose-Derived Stromal/Stem Cell Isolation, Expansion, and Transduction

All protocols were approved by the institutional review board of the Centre de Recherche du CHU de Québec-Université Laval and informed written consent was obtained from the donors. Human ASCs (healthy female donor, age: 35, body mass index (BMI): 21.0 (weight in kilograms divided by the square of the height in meters)) were isolated and amplified according to our previously described protocol [35], following lipoaspiration of subcutaneous adipose tissues. The banked hASCs were then transduced with a luciferase lentiviral vector according to previously described protocols [36]. Briefly, third generation lentiviral particles were produced after a lipofectamine 2000 co-transfection of pLenti CMV Puro LUC (Plasmid #17477; Addgene, Watertown, MA, USA) containing the luciferase gene under a cytomegalovirus (CMV) promoter, packaging pLP1, pLP2, and envelope pLP/VSVG (Addgene) plasmids into HEK293FT cells (Invitrogen, Burlington, ON, Canada). Lentiviral vectors containing cell culture supernatants were harvested every 12 h over three days, buffered to a pH of 7.4 using 1 × 10^−2^ M 4-(2-hydroxyethyl)-1-piperazineethanesulfonic acid (HEPES), and stored at 4 °C until being filtered using a 0.45 µm syringe filter and concentrated by ultracentrifugation at 5 × 10^4^ *g* for 90 min. Lentiviral stocks were made after resuspending pellets in fresh culture media and stored at −80 °C until use. Human ASCs (Passage 1) were transduced with luciferase lentiviral vectors in the presence of DEAE-dextran (10 µg mL^−1^) to increase transduction efficiency. Near confluency, the cells were passaged (split ratio = 1:4 after seven days) and placed under puromycin selection (1 µg mL^−1^). Then, these luciferase-transduced hASCs (hASCs-Luc) were frozen and banked in liquid nitrogen. For the production of all the substitutes, cryopreserved hASCs-Luc (Passage 5) were expanded (split ratio = 1:4 after seven days) in Dulbecco’s modified Eagle’s medium (DMEM) supplemented with fetal calf serum (FCS) (10%; Hyclone, GE Healthcare, Wauwatosa, WI, USA), and antibiotics (100 U mL^−1^ penicillin (Sigma-Aldrich, Oakville, ON, Canada) and 25 µg mL^−1^ gentamicin (Schering-Plough Canada Inc./Merck, Scarborough, ON, Canada)), and cultured in a humidified 37 °C incubator with CO_2_ (8%).

### 2.2. Human Umbilical Vein Endothelial Cell Isolation, Transduction, and Expansion

HUVECs were isolated from an umbilical cord by enzymatic digestion with thermolysin solution (250 µg mL^−1^; Sigma-Aldrich), as previously described [37,38]. Green fluorescent protein (GFP)-lentiviral vector particles were produced and purified, similarly to the luciferase vectors described above, after co-transfection of pLenti6.3/V5/EMGFP (Invitrogen), pLP1, pLP2, and pLP/VSVG plasmids with lipofectamine 2000 in HEK293FT cells. Briefly, the HUVECs (Passage 1) were transduced for six hours with the GFP lentiviral vectors in the presence of polybrene (8 µg mL^−1^; Sigma-Aldrich) before changing the media. Three days later, nearly confluent HUVECs were trypsinized and then sorted for GFP-positive cells (greater than 95% positive) using a FACSCalibur sorting module (BD Biosciences, Mississauga, ON, Canada), before expanding (split ratio = 1:6 after four days) and banking cell stocks. For the production of tissues used in the in vitro and in vivo experiments, both GFP-labeled and non-GFP HUVECs, respectively, were expanded (Passage 4; split ratio = 1:6 after four days) and cultured in endothelial growth medium-2 microvascular (EGM-2 MV) associated with BulletKit^TM^ (Lonza, Walkersville, MD, USA), in a humidified 37 °C incubator with CO_2_ (8%).

### 2.3. Production of Human Prevascularized Bone-Like Substitutes

**Figure 1:**
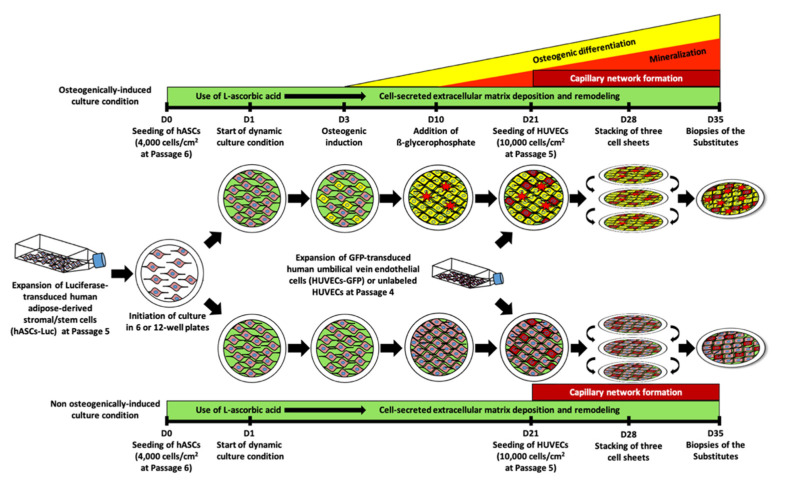
Schematic of the self-assembly approach of tissue engineering applied to the production of cell-derived prevascularized bone-like substitutes. Banked human adipose-derived stromal/stem cells (hASCs) were expanded at Passage 5 for a week. Subsequently, cell-secreted extracellular matrix deposition (green rectangles) was initiated by seeding hASCs at Passage 6 in 6- or 12-well plates at day 0 (D0), which were exposed to L-ascorbic acid stimulation until the day of the biopsies (D35). A dynamic wave-like movement was started one day after seeding the expanded hASCs (D1) and continued until the end of the culture period (D35) to favor cell sheet formation. However, to allow cell adhesion, the dynamic culture was stopped for 24 h, following endothelial cell seeding (D21). Osteogenic induction (yellow triangle) was started at day 3 (D3) to achieve osteoblastic differentiation and mineralization (red triangle). Expanded endothelial cells (HUVECs) were seeded (Passages 4 to 5) on top of the cell sheets at day 21 (D21) and spontaneously formed capillary networks (red rectangles) within one week. Finally, three prevascularized cell sheets containing capillary networks were stacked together at day 28 (D28) and cultured for an additional week to create a prevascularized bone-like tissue. Using the same process of self-assembly, prevascularized and non-prevascularized stromal tissues were produced from hASCs in the absence of osteogenic stimulation and served as controls.

Luciferase-transduced hASCs were seeded at a density of 4 × 10^3^ cells cm^−2^ (Passage 6) in 6- or 12-well plates containing an annular anchorage device (Whatman paper, Fisher Scientific, Quebec City, QC, Canada) (Figure 1). Cells were grown in DMEM, including FCS (10%), and supplemented with 1.8 × 10^−3^ M calcium chloride (CaCl_2_; Sigma-Aldrich). Media were changed three times a week, to which, a freshly prepared solution of sodium L-ascorbic acid (50 μg mL^−1^; Sigma-Aldrich) was added. Additionally, the cultures were exposed to wave-like dynamic movement using an orbital platform (Model 260301F, Ocelot, Fisher Scientific) set at 35 rpm, beginning one day (D) after seeding (D1) and continuing until stacking of the resulting cell sheets (D28). The dynamic movement was stopped for a 24 h period for all conditions on day 21 when endothelial cells were seeded. After 28 days of culture (D28), cell sheets were manually lifted from standard culture plastic plates using sterile forceps. Handling the cell sheets using the annular anchorage device preserves the cell sheet structure, as previously described [15]. Then, three cell sheets were stacked and clipped together to produce a multilayer 3D construct. In order to favor a strong cohesion between the cell sheet layers, the constructs were maintained in culture for seven additional days during which ascorbic acid further stimulated cell-secreted ECM deposition.

After three days of culture (D3), hASCs were osteogenically-differentiated by supplementing the complete DMEM containing fresh sodium L-ascorbic acid with osteogenic inducers composed of 10 × 10^−9^ M dexamethasone, 10 × 10^−9^ M 1α,25-dihydroxyvitamin D3, 50 × 10^−6^ M ascorbate-2-phosphate, and 1.8 × 10^−3^ M calcium chloride (osteogenic medium; Table 1). Additionally, osteogenic medium was supplemented with 3.5 × 10^−3^ M β-glycerophosphate beginning at culture day 10 (D10) to initiate biomineralization. All described supplements were purchased from Sigma-Aldrich. After 21 days of culture (D21), GFP-labeled (for in vitro experiments) or unlabeled HUVECs (for in vivo experiments) were seeded at a concentration of 1 × 10^4^ cells cm^−2^ (Passage 5) on the surface of the cell sheets (Figure 1). After the addition of the endothelial cells, the tissues were cultured in a 1:1 ratio of the respective complete media and EGM^TM^-2 MV media (Lonza) until the end of culture (Table 1), as previously described [34]. Upon reconstitution of the 1:1 combined medium, the final concentration of pro-angiogenic growth factors was diluted by two, while the concentration of osteogenic factors was maintained by doubling their initial concentration (Table 1). Then, three prevascularized cell-sheets were superimposed and cultured for seven additional days to favor the development of capillary networks and to generate a prevascularized bone-like substitute. Non-osteogenically-differentiated hASCs were cultured in complete DMEM medium, without osteogenic inducers or β-glycerophosphate, to generate prevascularized stromal substitutes (basal medium; Table 1). In this study, non-prevascularized bone-like and stromal substitutes were cultured in osteogenic and basal media, respectively, and used as controls.

### 2.4. Two-Dimensional Characterization of Capillary Network Formation

The organization of HUVECs into capillary structures at the surface of individual cell sheets was evaluated daily for six days after seeding GFP-transduced HUVECs. Stitched images of the entire area of the tissues were taken using a Zeiss fluorescence microscope equipped with a Colibri 7 LED illumination system (Zeiss, Toronto, ON, Canada). Central regions of interest (ROI) (25 mm^2^) of the stitched pictures were then analyzed using the Angiogenesis Analyzer plug-in of ImageJ^®^ software (NIH, Bethesda, MD, USA), as previously described [39]. GFP-positive structures such as master segments, length of the master segments, meshes, and master junctions were analyzed for each condition (n = 3 tissues/condition per time point).

### 2.5. Histological Analyses of Substitutes and Explanted Tissues

Histological analyses were performed on reconstructed tissues after 35 days of culture (D35), as well as 12 weeks after grafting on explanted tissues. Samples of each condition were fixed in 3.7% formaldehyde overnight at room temperature (RT). Skull tissues containing the implantation sites were demineralized using a 0.6 N hydrochloride (HCl) solution for 48 h at 4 °C. Tissues were then paraffin-embedded and five-micrometer thick cross-sections were cut. Samples were stained with Masson’s trichrome. All images represent stained cross-sections, and unless otherwise stated, were captured under brightfield illumination using a Zeiss Observer Z1 inverted microscope (Zeiss) equipped with an AxioCam ICc1 camera.

### 2.6. Human CD31 Indirect Immunofluorescent Labeling on Substitute’s Cross-Sections

Indirect immunofluorescent labeling for human CD31 was performed on tissue samples harvested after 35 days of culture. Samples were embedded in optimum cutting temperature (OCT) compound (Tissue-Tek OCT, Sakura Finetek USA Inc., St. Torrance, CA, USA), and frozen at −80 °C prior to sectioning. A cryostat was used to obtain cryosections (10 μm) that were fixed in formaldehyde (3.7%) for 20 min at RT. Afterward, samples were washed in phosphate buffered saline (PBS) and incubated for one hour at RT with a sheep anti-human CD31 primary antibody (AF806; 1:100, R&D Systems, Minneapolis, MN, USA) diluted in blocking solution (1X PBS/5% bovine serum albumin (BSA)/0.3% Triton). The samples were then incubated with an Alexa 633-conjugated donkey anti-sheep antibody (A21100, 1:500, Life Technologies, Woburn, MA, USA) for 30 min at RT protected from light. Hoechst solution (0.5 µg mL^−1^; Sigma-Aldrich) was used in parallel to label cell nuclei. Finally, samples were covered with mounting medium (1X PBS/2% gelatin/15% glycerol) and glass coverslips and stored at 4 °C prior to imaging. Images were captured using an LSM 700 confocal microscope (Zeiss) and equally color-balanced using Zen 2010 software (Zeiss).

### 2.7. Transmission Electron Microscopy of Capillary Networks in Substitutes

The ultrastructure of the capillary structures found in reconstructed tissues was observed after 35 days of culture. Samples were fixed and processed for transmission electron microscopy (TEM) analysis, as described previously [40]. Briefly, samples were fixed for 24 h at 4 °C in glutaraldehyde (2.5%; Canemco & Marivac, Lakefield, QC, Canada). Then, samples were washed with cacodylate buffer (0.1 M; Mecalab, Montreal, QC, Canada), and post-fixed with osmium tetroxide (1%) for 90 min at RT. Finally, ultrathin sections stained with uranyl acetate were imaged using a JEOL JEM 1230 transmission electron microscope (JEOL, Tokyo, Japan) at a voltage of 80 kV. Samples from two separate experiments were imaged for each condition (n = 1 tissue/condition).

### 2.8. CD31 Immunolabeling and 3D Network Reconstruction in Prevascularized Substitutes

In toto labeling of samples was performed after 35 days of culture for the detection of the CD31 endothelial cell marker. The entire anchored prevascularized tissues were fixed in formaldehyde (3.7%) for 20 min and washed using PBS. Afterward, tissues were incubated overnight at 4 °C with a sheep anti-human CD31 primary antibody (AF806; 1:100, R&D Systems) diluted in blocking solution (1X PBS/5% BSA/0.3% Triton). Following a second washing step, tissues were incubated with Alexa 633-conjugated donkey anti-sheep antibody (A21100, 1:500, Life Technologies) for two hours at RT. Tissues were mounted with a coverslip. CD31 positive structures were imaged using Z-stack confocal microscopy (LSM700, Zeiss). The 3D reconstruction and the analysis of the capillary networks were generated using Imaris^®^ software (Bitplane, Concord, MA, USA) [41,42].

### 2.9. Enzyme-Linked Immunosorbent Assay for Human Osteocalcin in Substitute’s Extracts

The presence of the osteogenic marker osteocalcin was analyzed in extracts from the substitutes cultured for 35 days, as well as for tissues harvested from the animals 12 weeks after implantation, as previously described [33]. Briefly, flash-frozen tissues harvested for each condition were cryomilled using a Mixer Mill MM 400 (Retsch, Mann, Germany). Then, cryomilled tissues were resuspended at 20% (weight/volume) in 250 × 10^−3^ M Tris/0.5% Triton-100 lysate buffer with cOmplete^TM^ protease inhibitor (Sigma-Aldrich), and sonicated three times on ice. Human osteocalcin in whole tissue lysates was quantified by ELISA (KAQ1381, Thermo-Fisher Scientific, Mississauga, ON, Canada) according to the manufacturer’s instructions. Samples were run in duplicate for each condition (n = 4 tissues/condition).

### 2.10. O-Cresolphthalein Complexone Method for Calcium Quantification

The amount of calcium deposited in the matrix of the substitutes after 35 days of culture was quantified using an *o*-cresolphthalein complexone method [43]. Briefly, 5 mm diameter punch biopsies of the substitutes were collected for each group. Samples were decalcified in 0.6 N HCl (1 mL) for 48 h at 4 °C. After brief mixing and centrifugation, each extract supernatant (100 μL) was mixed with an ammonium chloride buffer (2.8% NH_4_OH, 24.9 × 10^−3^ M NH_4_Cl, pH 10.5), 8-quinolinol (0.22%), and o-cresolphthalein complexone solution (0.022%) (Sigma-Aldrich). The level of calcium in each sample was determined from a standard curve of known calcium concentrations measured at 575 nm (SPECTRA Plus 384, Molecular Devices). Samples were run in duplicate for each condition (n = 3 tissues/condition), and data were normalized to the punch biopsy area.

### 2.11. Fluorescent Hydroxyapatite Labeling, Imaging, and Quantification

Specific hydroxyapatite fluorescent labeling was performed after 35 days of culture on the whole substitutes that were fixed overnight at 4 °C in formaldehyde (3.7%). Hydroxyapatite was labeled with the Xenolight RediJect Bone Probe 680 diluted with demineralized water (1:50; Caliper Life Sciences, Hopkinton, MA, USA) for two hours at RT in the dark. Then, the substitutes were washed using PBS prior to imaging and fluorescent images were excited at 633 nm and captured at 705 nm (bandpass 40) using a Typhoon^TM^ Trio+ fluorescent scanner (GE Healthcare). The intensity of the fluorescent probe within the total area of the substitute (approximately 1.25 cm^2^) was quantified using ImageJ^®^ software for each condition (n = 4 tissues/condition).

### 2.12. Calvarial Bone Defect Surgery and Grafting of the Substitutes

Seventeen immunocompromised NIH-Foxn1 RNU nude rats (males, 12 weeks old; Charles River, Saint-Constant, QC, Canada) were used in this study according to the protocol approved by the Institutional Animal Protection Ethics committee of Université Laval (Protocol approval number: 2011-182). Under general anesthesia using isoflurane inhalation and local injection of lidocaine/bupivacaine (3.5 mg kg^−1^), two (bilateral) calvarial defects (5 mm diameter) were created in the parietal bones of each rat using a surgical drill. Depending of the animal, calvarial bones were approximately 1 mm thick. Calvarial bone defects were completely filled with the substitutes of either human prevascularized bone-like tissues, human prevascularized stromal tissues, human non-prevascularized bone-like tissues, or human non-prevascularized stromal tissues previously cultured for 35 days in vitro (n = 6 per experimental group). The substitutes were shaped using a 5 mm punch on each construct consisting of three fused cell sheets. Multiple punches were stacked directly into the calvarial defects and no membrane was necessary to maintain the substitutes in place. The number of punches necessary to fill the defect varied between groups depending on the substitute thickness (Figure S1) and the defect height. Untreated defects (empty) and rat skull bone, re-grafted from the calvarial defect and thus representing an autograft, were used as control groups (n = 3 per control group). The grafted tissues were analyzed 12 weeks after implantation.

### 2.13. Micro-Computed Tomography Imaging and Analysis

Terminal analyses were performed 12 weeks after implantation using micro-CT with a GE eXplore CT 120 scanner (GE Healthcare). The implantation sites were imaged using standard conditions (tube voltage: 100 kV, tube current: 50 mA). The 3D rendering and the bone volume analysis of the cylindrical ROI (5 mm diameter × 3 mm height) were performed using the Imeka^®^ MI (Medical Imaging) software (Imeka Solutions, Sherbrooke, QC, Canada). Sample data were calibrated against the densities of reference materials (air, water, and hydroxyapatite) used to generate a standard curve: air: −1000 Hounsfield units (HU), water: 0 HU, and bone (hydroxyapatite): +1550 HU. Bone volume was calculated as a percentage of measured native rat skull bone controls (100%). All analyses were performed using the same threshold level (700) to determine the limit between bone and soft tissues (n = 6 grafts/condition and n = 3 for controls).

### 2.14. Analysis of Graft Survival Using In Vivo Imaging System (IVIS)

Since hASCs-Luc were used to produce all the tissues in this study, their survival after 12 weeks of implantation was evaluated using an IVIS^®^ Lumina II device (Perkin Elmer, Waltham, MA, USA). Before and during imaging, animals were anesthetized using isoflurane inhalation. Luciferin was injected intraperitoneally (150 mg kg^−1^) and bioluminescent images were captured during a biodistributed saturation time of 15−30 min after luciferin injection. When in contact with the luciferase, the luciferin emitted bioluminescence (photons) that was detected and measured using a CCD camera cooled to −90 °C. The intensity of the bioluminescence indirectly indicates the viability of the cells composing the grafts. Images were acquired and analyzed using the Living Image software (Perkin Elmer) (n = 6 grafts/condition).

### 2.15. Statistical Analyses

Mean differences between groups were evaluated by performing unpaired Student’s t-tests, as well as one-way or two-way ANOVA with a Bonferroni’s multiple comparison post-hoc test using GraphPad Prism software (version 7, La Jolla, CA, USA). Where applicable, outliers were identified using Grubb’s method (Alpha = 0.1) and removed from data sets. All data are expressed as the mean ± standard deviation (SD), and differences with a *p* < 0.05 were considered significant.

## 3. Results

### 3.1. Production of All-Natural and Human Prevascularized Bone-Like Tissues

Prevascularized bone-like tissue constructs were generated in vitro using a cell sheet technology (Figure 1). These human cellularized tissues consist of all-natural biomaterials including a spontaneously formed capillary network of endothelial cells. They result from the self-assembly of human extracellular matrix components secreted and assembled by hASCs stimulated with ascorbic acid and osteogenic inducers (Table 1). The cellular support is generated in vitro directly by the cells (hASCs) over a long-term culture period. This approach leads to osteogenically-differentiated cell sheets that are then superimposed and allowed to fuse into thicker tissues. Final construct thickness varied between bone-like and stromal substitutes, with bone-like tissues presenting values of 150 µm (+HUVEC) to 185 µm (−HUVEC) (Figure S1). This natural biofabrication process generates a biomimetic construct that is entirely biological and human-based, without the use of external cross-linkers or chemically modified materials. The constructs can easily be handled using a peripheral anchorage device (Figure 1), which is removed before in vivo implantation.

### 3.2. Kinetics of Capillary Network Organization on Prevascularized Cell Sheets

**Figure 2:**
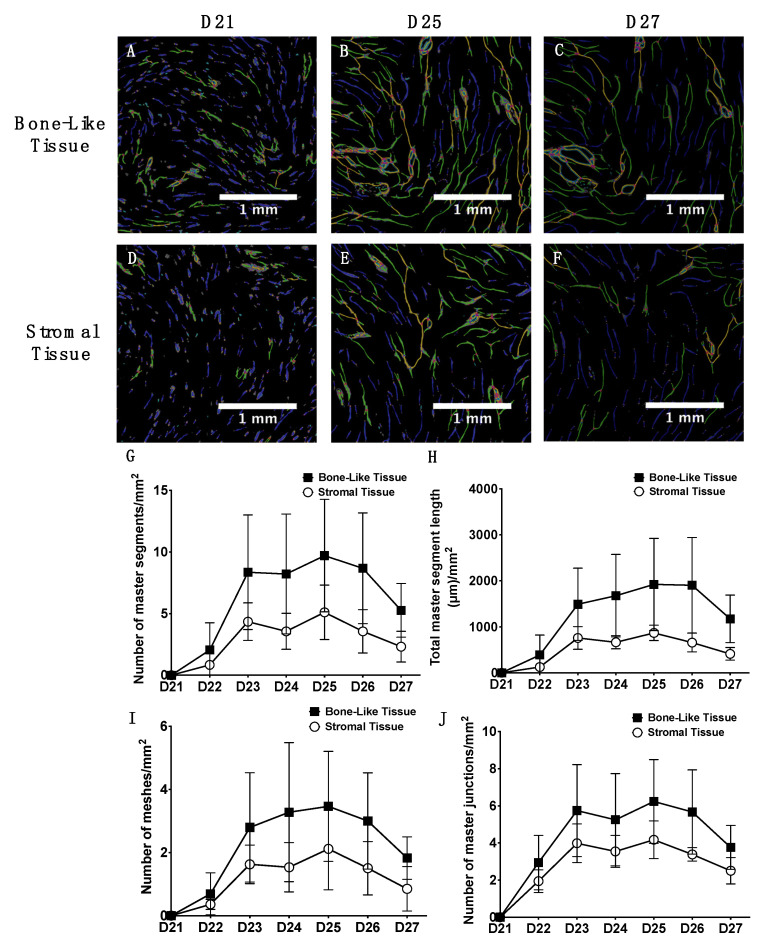
Kinetics of the capillary network formation on individual cell sheets over six days of maturation, prior to cell sheet stacking into tissues. Large regions of interest of the prevascularized cell sheets were imaged for GFP-positive structures for six consecutive days (D21 through D27) after endothelial cell seeding (D21) (**A**–**F**). Pictures were then analyzed using the Angiogenesis Analyzer plug-in of ImageJ^®^ software. Scale bars, 1 mm. Temporal analysis of in vitro capillary network formation revealed more numerous (but non-significant): numbers of master segments per mm^2^ (yellow lines in (**A**–**F**)) (**G**), length of the master segments per mm^2^ (**H**), number of meshes per mm^2^ (cyan blue meshes in (**A**–**F**)) (**I**), and master junctions (pink dots in (**A**–**F**)) connecting two master segments per mm^2^ (**J**) within cell sheets induced towards osteogenesis compared to stromal cell sheets. Two-way ANOVA with a Bonferroni’s multiple comparisons test (n = 3 tissues/condition per time point).

The development and organization of the capillary networks were investigated for six consecutive days, beginning one day after the seeding of endothelial cells (Figure 2: D21 to D27). Green fluorescent protein (GFP) expression from HUVECs allowed the evaluation of the kinetics of capillary formation on individual living cell sheets. Imaging of the structures showed the development of a well-organized network on both the osteogenically-induced and the stromal cell sheets (Figure 2). Capillary networks were evaluated for the presence of master segments (yellow lines) composed of three segment pieces (blue lines for individual segments) delineated by two master junctions (pink dots) that are non-exclusively implicated with one branch (green lines), and meshes (cyan blue meshes) that are enclosed by segments or master segments (Figure 2A–F). Quantitative analyses of the GFP-positive structures of the networks revealed a greater but non-statistically significant increase in the number of master segments (Figure 2G), total master segment length (Figure 2H), meshes (Figure 2I), and master junctions (Figure 2J) for the osteogenic-induced cell sheets compared to the stromal ones over time. These results show that HUVECs can spontaneously organize into a capillary network after only four days at the surface of the cell sheets. Moreover, after four days, the reorganization of the networks is suggested by a decrease in plateau levels for both types of tissues (Figure 2G–J).

### 3.3. Characterization of Capillary-Like Morphological Features in Prevascularized Tissues

**Figure 3:**
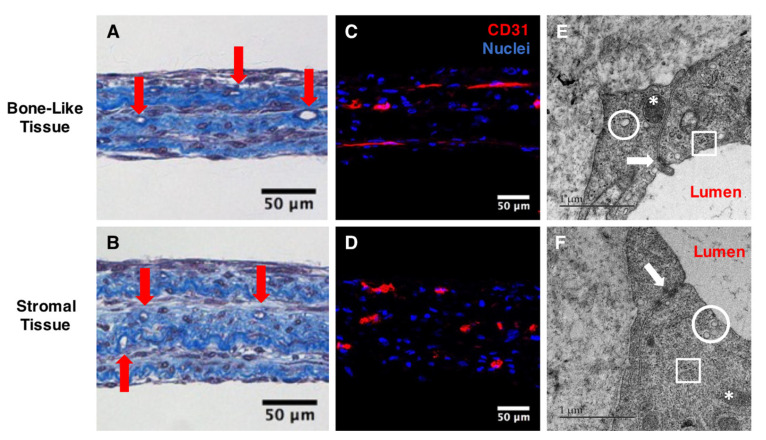
Characterization of the capillary network in prevascularized substitutes after two weeks of HUVEC network maturation. Masson’s trichrome staining of transverse sections of prevascularized tissues revealed the presence of lumen-like structures (red arrows) in both bone-like (**A**) and stromal tissues (**B**). Scale bars, 50 µm. CD31-positive structures (red) were observed in both tissue types after the immunolabeling of cryosections (**C**,**D**). Nuclei were stained in parallel with Hoechst (blue). Scale bars, 50 µm. Transmission electron microscopy images show typical ultrastructural features of blood capillaries, such as caveolae (white circles), Weibel–Palade bodies (white asterisks), tight junctions (white arrows), glycogen inclusions (white squares), and lumens (**E**,**F**). Scale bars, 1 µm.

The characteristics of the capillary networks within prevascularized 3D reconstructed substitutes, each comprising three cell sheets, were evaluated after 35 days of culture (14 days after endothelial cell seeding). Histological observations after Masson’s trichrome staining revealed the presence of lumen-like structures in both bone-like and stromal tissues (Figure 3A,B). Immunostaining for CD31 confirmed that these lumen-like structures were derived from the human endothelial cells incorporated within both tissue types (Figure 3C,D). Bone-like tissues and stromal controls without HUVECs were immunolabeled for CD31, confirming that hASC cultures did not contain subpopulations capable of generating endogenous capillary networks under these culture conditions (Figure S2). Transmission electron microscopy observations validated the presence of hallmark features of microvascular structures among endothelial-lined lumens, including the presence of caveolae (white circles), Weibel–Palade bodies (white asterisks), glycogen inclusions (white square), and tight junctions (white arrows) (Figure 3E,F).

### 3.4. Three-Dimensional Reconstruction Following Imaging to Evaluate Capillary Network Segments and Volume/Density within the Substitutes

**Figure 4:**
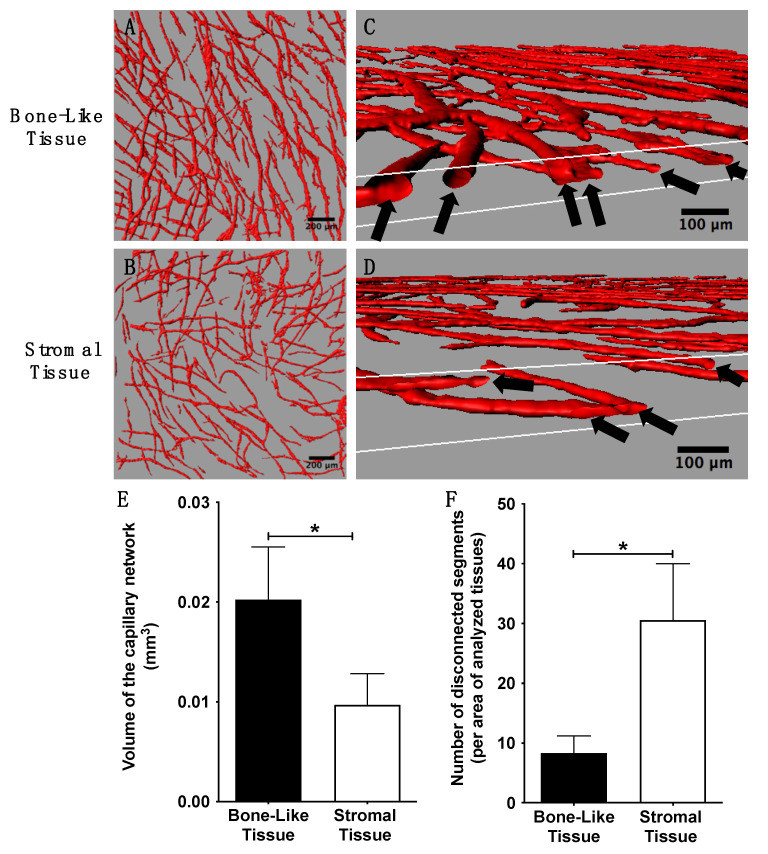
Characterization of the capillary network in reconstructed tissues two weeks after the seeding of endothelial cells. Three-dimensional reconstructions of CD31 positive structures (red) using Imaris^®^ software (**A**–**D**). Top views of the 3D reconstructions show greater capillary network density in bone-like tissues (**A**) compared to stromal tissues (**B**). Scale bars, 200 µm. Cross-sectional views of the 3D capillary networks revealed the presence of capillary lumens (black arrows) (**C**) and (**D**). Scale bars, 100 µm. Evaluation of the 3D reconstructed capillary network showing that bone-like tissues, when compared to stromal tissues, featured a significantly larger tissue volume occupied by CD31 positive structures (**E**), as well as a more connected capillary network, as shown by the lower number of disconnected components (**F**). Unpaired *t* tests, * *p <* 0.05.

The global appearance of the capillary networks within the reconstructed substitutes was determined by assessing CD31 positive structures using confocal imaging and software analysis. These networks were reconstructed in 3D to evaluate the overall coverage of the reconstructed substitutes, as well as the degree of connections between capillary structures. Observations revealed a greater capillary network density within the bone-like tissues compared to the stromal ones (Figure 4A,B). The presence of lumens was confirmed in both tissue types (Figure 4C,D). Quantitative analyses showed the presence of a significantly elevated volume of CD31 positive structures (2.1-fold; *p* < 0.05; Figure 4E), as well as a lower number of disconnected segments (3.7-fold; *p* < 0.05; Figure 4F) in the bone-like tissues compared to the stromal tissues. Taken together, these results indicate that after 14 days of in vitro remodeling, the reconstructed bone-like substitutes featured a dense and extended network of capillaries.

### 3.5. In Vitro Matrix Deposition of Osteocalcin within the Substitutes

**Figure 5:**
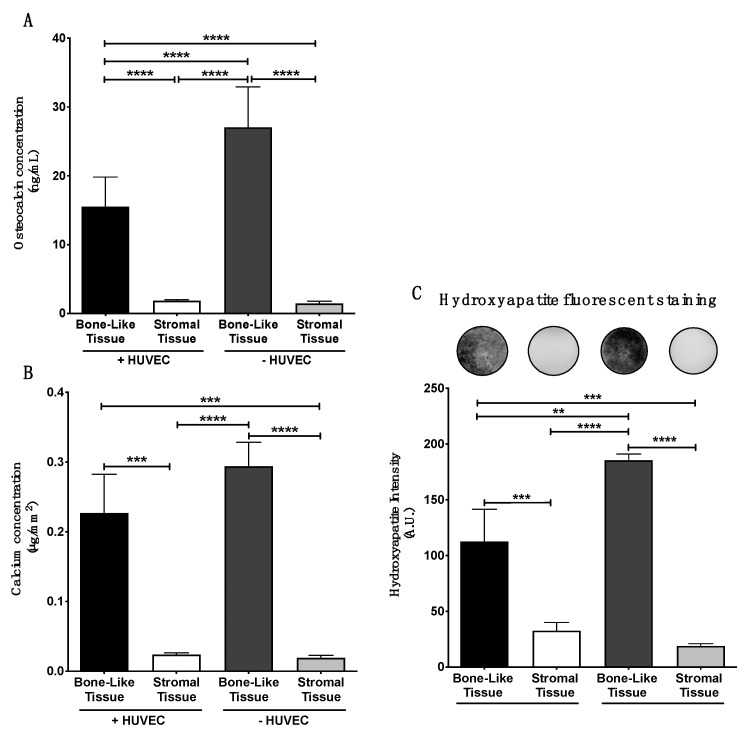
Adding HUVECs diminishes in vitro osteogenesis and biomineralization of the bone-like tissues analyzed after 35 days in culture. The level of human osteocalcin detected by ELISA from tissue lysates is significantly reduced in the prevascularized bone-like tissues (**A**). Calcium content in osteogenically induced tissues, in presence or absence of HUVECs, was significantly elevated compared to their stromal counterparts (**B**). Fluorescent staining of hydroxyapatite in whole tissues was imaged and quantified using a Typhoon Trio+ scanner (**C**). Fluorescent intensity was significantly elevated in bone-like tissues, in presence or absence of HUVECs, compared to their stromal tissue counterparts. One-way ANOVA with a Bonferroni’s multiple comparisons post-hoc test, ** *p* < 0.01, *** *p* < 0.001, **** *p* < 0.0001.

Late stages of osteogenic differentiation of the reconstructed tissues were assessed by determining OCN protein presence in the lysates of tissue extracts after 35 days of culture. Supplementation with standard osteogenic agents in complete media induced and promoted osteogenic differentiation of hASCs. Indeed, significantly elevated levels of OCN were quantified in the bone-like tissues compared to stromal tissues in absence of endothelial cells (19-fold; *p* < 0.0001; Figure 5A, −HUVEC). Furthermore, prevascularized bone-like tissues contained increased quantities of OCN compared to prevascularized stromal tissues (8.6-fold; *p* < 0.0001; Figure 5A, +HUVEC). However, significantly reduced amounts in OCN were quantified in prevascularized bone-like tissues compared to their non-prevascularized counterparts (1.7-fold; *p* < 0.0001, Figure 5A). These results show that while osteogenesis is initiated by supplementation with osteoinducers, the addition of endothelial cells at day 21 of culture in osteogenic-induced substitutes appears to impede osteocalcin deposition within the matrix of the reconstructed bone-like tissues, when assessed 14 days later.

### 3.6. Matrix Mineralization within the Bone-Like Tissues

In vitro evaluation of tissue biomineralization was performed on the reconstructed substitutes at the end of the culture period (D35). Independently of HUVEC presence, the quantification of calcium within the matrix was significantly higher in the bone-like tissues compared to either of the stromal tissues (Figure 5B). Calcium levels quantified between the prevascularized and the non-prevascularized bone-like tissues did not differ significantly. Measurement of hydroxyapatite probe intensity showed a significantly elevated signal within the bone-like tissues compared to the stromal ones, independently of HUVEC presence (Figure 5C). Nevertheless, the intensity of the fluorescent probe was higher in non-prevascularized bone-like tissues compared to their prevascularized counterparts (1.6-fold, ** *p* < 0.01) (Figure 5C). These results suggest that the addition of a prevascularization step during tissue production may reduce matrix mineralization in vitro after 35 days of culture.

### 3.7. Healing of Calvarial Bone Defects in a Rodent Model

**Figure 6:**
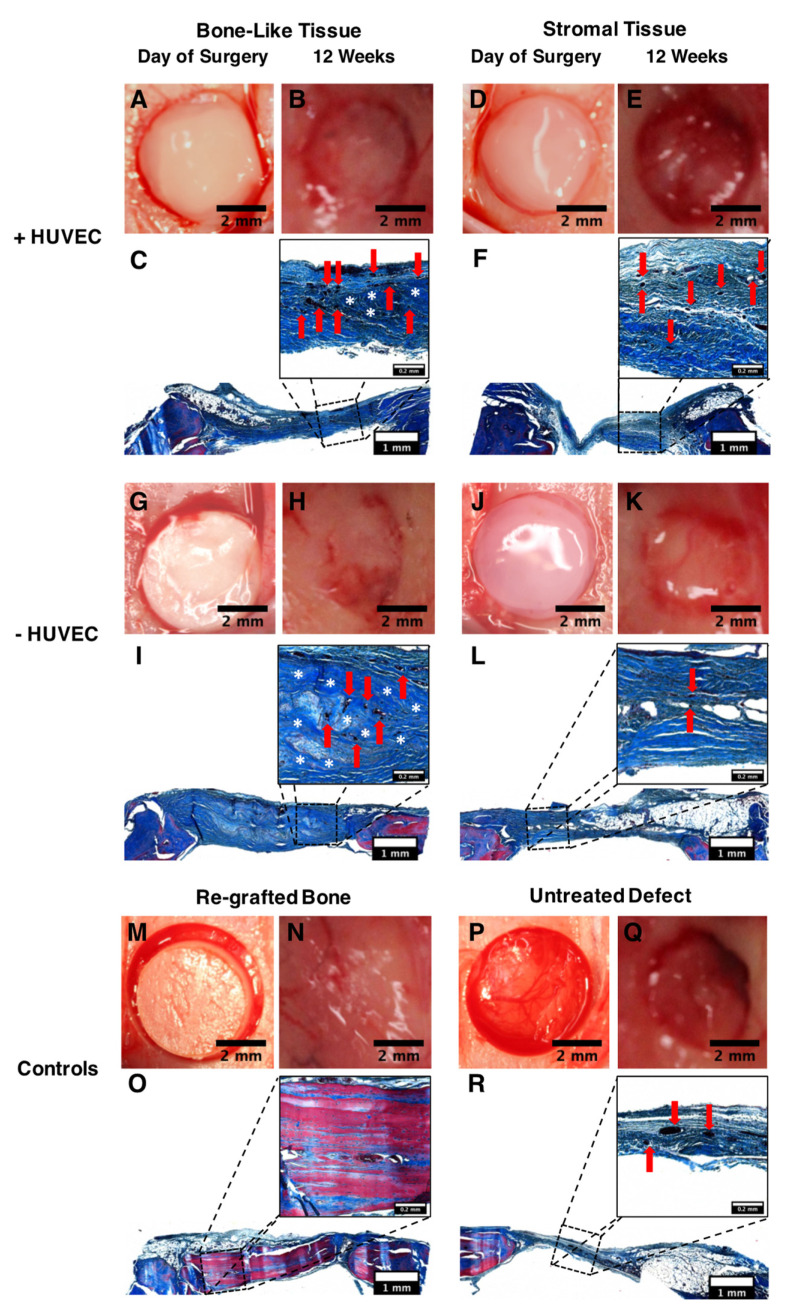
Rat calvarial bone defects grafted with the various types of tissues. Macroscopic images and histological cross-sections of the implantation sites on the day of surgery and 12 weeks after grafting (**A**–**R**). Bone defects filled with the reconstructed bone-like tissues and their associated histological features (**A**–**C**) and (**G**–**I**) compared to defect sites filled with the reconstructed stromal tissues (**D**–**F**) and (**J**–**L**). Re-grafted native rat calvarial bone (**M**–**O**) and untreated defect groups were used as references (**P**–**R**). Capillary blood vessels (red arrows), as well as new bone formation (white stars, intense blue) are visible at higher magnification (insets). Scale bars, (**A**,**B**,**D**,**E**,**G**,**H**,**J**,**K**,**M**,**N**,**P**,**Q**) 2 mm and (**C**,**F**,**I**,**L**,**O**,**R**) 1 mm and 0.2 mm (insets).

Substitutes composed of stacked cell sheets were matured for 35 days in vitro before their implantation into 5 mm calvarial bone defects. Macroscopic images show the positioning of the substitutes in the defects at the time of surgery (Figure 6A,D,G,J), as well as the controls used, consisting of rat native bone regrafted into the defects (Figure 6M), as well as empty defects (Figure 6P). Twelve weeks after grafting, macroscopic images of the implantation sites grafted with bone-like tissue constructs (+HUVECs and −HUVECs) revealed improved healing compared to stromal tissues. This is also supported by the observations made on histological tissue cross-sections of increased new bone tissue and matrix formation (intense blue and red), with less connective tissue presence (light blue) when defects are grafted with bone-like tissue constructs, independently of prevascularization (Figure 6C,F,I,L,O,R). Similar to the re-grafted native bone (Figure 6M–O), all of the reconstructed tissues were integrated into the surrounding native tissue. In addition, no signs of infection or inflammation were observed. Higher magnification of the grafted areas showed the presence of capillary blood vessels (red arrows) and new bone formation (intense blue; white stars) at the core of the implantation sites that were grafted with bone-like tissue constructs enriched or not with HUVECs (Figure 6C,I).

### 3.8. Micro-Computed Tomography Analysis and Reconstruction of the Grafted Bone Defects

**Figure 7:**
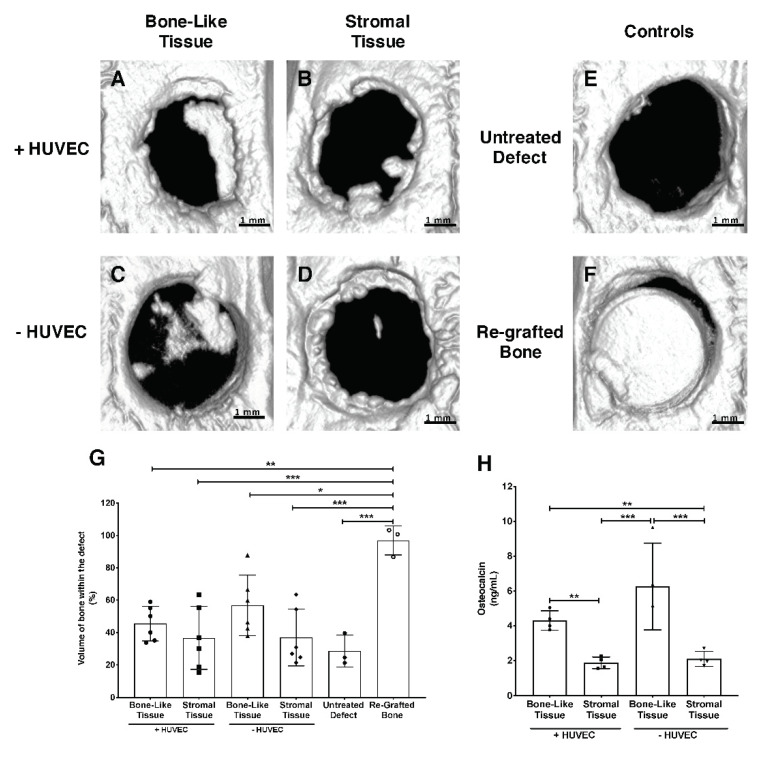
Micro-CT analysis of calvarial bone defect sites 12 weeks after tissue implantation and quantification of osteocalcin in explanted tissues. Micro-CT images of the bone defects treated with reconstructed bone-like or stromal tissues, in presence or absence of prevascularization (**A**–**D**). Untreated defect (**E**) and re-grafted bone (**F**) groups were used as controls to mimic the lack of intervention, or an autograft procedure, respectively. The bone is represented by the white structures on a black background at a threshold of 700. Scale bars, 1 mm. The percentage of bone volume at the implantation sites was quantified based on micro-CT scans (**G**). Osteocalcin quantification in tissue lysates from the explanted defect sites (**H**). Twelve weeks after implantation, prevascularized (+HUVEC) and non-prevascularized (−HUVEC) bone-like tissue constructs contained elevated levels of human osteocalcin compared to stromal tissues but were not different from each other. Native rat bone was used to assess nonspecific binding between species for osteocalcin, which was considered background. One-way ANOVA with a Bonferroni’s multiple comparisons test, * *p* < 0.05, ** *p* < 0.01, *** *p* < 0.001.

Bone repair assessment (terminal analysis) was performed based on micro-computed tomography (micro-CT) scans of the animals 12 weeks after grafting. Three-dimensional renderings of the scans showed increased mineralized tissue formation in the defects that were grafted with the bone-like tissue substitutes (Figure 7A,C) compared to those grafted with the stromal substitutes (Figure 7B,D), independently of prevascularization. Based on micro-CT data quantification, the mean volume of new bone formed within the defect sites did not vary significantly among the groups implanted with the reconstructed tissues (Figure 7G). In this experiment, the re-grafted bone group (Figure 7F) was used to mimic an autograft procedure and acted as a reference. When compared to this reference group, the non-prevascularized and prevascularized bone-like tissues showed less (1.7-fold; *p* < 0.05 and 2.1-fold; *p* < 0.01, respectively) empty bone volume at the implantation sites compared to those grafted with prevascularized or non-prevascularized stromal tissues (2.6-fold; *p <* 0.001 and 2.6-fold; *p <* 0.001, respectively). These results suggest that the non-prevascularized and prevascularized bone-like substitutes have inherent qualities that may facilitate osteogenesis, with the potential to repair the defect site over time compared to their stromal counterparts in this model.

### 3.9. Human Osteocalcin Content in Tissues 12 Weeks after Implantation

The level of human osteocalcin deposited within the reconstructed substitutes at the defect sites was quantified 12 weeks after grafting. Levels in tissues from groups of animals treated with bone-like substitutes were not appreciably modulated by prevascularization (Figure 7H). However, osteocalcin levels measured for both of these explanted bone-like substitutes remained markedly elevated compared to animals treated with the stromal substitutes, irrespective of in vitro prevascularization (+HUVEC: 2.3-fold; *p* < 0.01 and −HUVEC: 3.3-fold; *p* < 0.001) (Figure 7H). These results suggest that prevascularized bone-like substitutes are permissive to osteocalcin deposition in the newly formed matrix in vivo at the treated defects over the 12-week period of healing.

### 3.10. Viability of Implanted Prevascularized Substitutes

**Figure 8:**
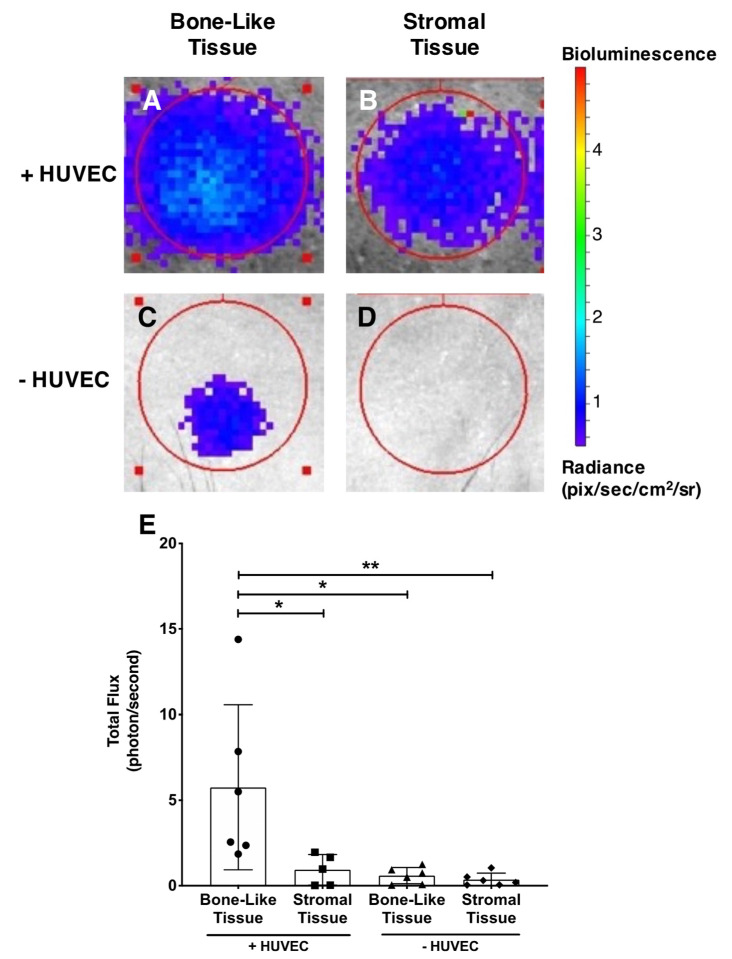
Functional analysis of hASC-Luc-derived cells in reconstructed substitutes after 12 weeks of implantation. The bioluminescence emitted by hASCs-Luc composing the grafted tissues was imaged 15 min after intraperitoneal luciferin injection using an IVIS^®^ Lumina II device (**A**–**D**). Red circles indicate both the size of the original 5 mm diameter bone defect and ROI for bioluminescent signal acquisition. The prevascularized (+HUVEC) bone-like substitutes emitted mean elevated levels of bioluminescence compared to prevascularized stromal tissues, as well as non-prevascularized substitutes (−HUVEC, bone-like and stromal) after 12 weeks of implantation (**E**). The normal distribution of the data set was validated with a Shapiro–Wilk test, and Grubb’s method (Alpha = 0.1) was used to identify and remove outliers. A one-way ANOVA was then used with a Bonferroni’s multiple comparisons post hoc test, * *p* < 0.05, ** *p* < 0.01.

The viability of the luciferase-transduced hASCs composing the substitute grafts was evaluated 12 weeks after implantation in living animals. Before IVIS imaging, the animals were given an intraperitoneal injection of luciferin. The cells in the grafted prevascularized bone-like substitutes emitted a significantly elevated bioluminescent signal compared to those in the prevascularized stromal substitutes (4.3-fold; *p* < 0.05; Figure 8), non-prevascularized bone-like substitutes (5.7-fold; *p* < 0.05), and non-prevascularized stromal substitutes (18.3-fold; *p* < 0.01; Figure 8A–E). By way of functional analysis, these results suggest an enhanced survival of initial hASCs incorporated into the grafted prevascularized bone-like tissues after 12 weeks in vivo.

## 4. Discussion

In the past years, cell sheet technologies have been revealed as useful models to generate biomimetic cellularized constructs for maxillofacial and craniofacial repair [15]. Among these technologies, the self-assembly approach is based on the capacity of mesenchymal cells to secrete and assemble their endogenously produced extracellular matrix components upon culture in the presence of ascorbic acid and serum, without the use of thermoresponsive culture plates [44,45]. Among the wide variety of biomaterials available for tissue reconstruction, a clear tendency towards the use of more natural scaffolding elements is seen. For example, improvements in decellularization protocols prompted a comeback of these matrix-rich natural biomaterials as support for reseeded cells, whether from bone or other tissues [46,47,48]. In our system, the self-assembly approach allows the secretion, assembly, and mineralization of ECM in a native state from living cells, bypassing the challenges associated with decellularization/recellularization processes. The present study shows that endothelial cells-derived capillary networks could spontaneously form in vitro during osteogenic tissue production based on the self-assembly approach. Indeed, the formation of dense and well-organized capillary-like networks was observed after two weeks of maturation in vitro. This was associated with reduced osteocalcin levels and hydroxyapatite formation in prevascularized bone-like tissues after 35 days of culture. These in vitro results differ from our initial hypothesis. However, upon implantation into calvarial bone defects, in vivo imaging of the prevascularized constructs indicated an improvement in cell viability in the grafts, without impeding bone healing after 12 weeks.

Prevascularization strategies in tissue engineering apply to several types of substitutes. Lovett et al. described that prevascularization has a critical role in the successful implantation of thick reconstructed tissue (>200 µm) [27]. The presence of a pre-existing microvasculature in engineered tissues may favor a rapid and adequate oxygen/nutrient supply by allowing a quicker anastomosis between the blood vessels of host and grafted tissues [32,49,50]. This may significantly increase graft survival [31,32,50] and favor the healing process. Native cortical bone is a complex tissue composed of a dense mineralized matrix, including the Haversian system that contains the bone microvasculature [51]. This microvasculature is essential for the maintenance of bone homeostasis, to remove metabolic wastes, and to supply oxygen and nutrients to the tissue. In bone tissue engineering, the interactions between osteoprogenitors and endothelial cells are important for the production of optimal bone substitutes. Pirraco et al. showed the potential of using HUVECs associated with rat bone-marrow mesenchymal stem cells (rBMSCs) osteogenic cell sheets produced using thermoresponsive plates to improve bone formation at ectopic sites in vivo [28]. Their study also showed the presence of human CD31 positive structures in the prevascularized tissues in vitro, as well as in vivo after grafting [28]. In the present study, we show the presence of an extensive network of endothelial capillaries in vitro when CD31 positive HUVECs are seeded into hASC-derived reconstructed tissues after 21 days of culture. Indeed, HUVECs spontaneously formed a well-organized capillary network featuring lumens during the 14 days of culture. At the ultrastructural level, the capillaries featured caveolae, Weibel–Palade bodies, glycogen inclusions, and tight junctions. Our results seem to suggest that the organization of the network is further favored when endothelial cells are surrounded by hASCs that are osteogenically differentiated. This could be explained by paracrine cell-to-cell communication between the two cell types [52]. Indeed, it was reported that vitamin D_3_ and ascorbate-2-phosphate, which are components of the osteogenic induction cocktail, in addition to endogenous bone morphogenetic proteins (BMPs) secreted by the endothelial cells, can promote the secretion of vascular endothelial growth factor (VEGF) by the osteoblasts [15,52,53,54]. Consequently, osteoblast-secreted VEGF production, in addition to the secretion of other mediators, could stimulate endothelial cell proliferation and tubulogenesis.

In our previous work, we showed that hASCs secrete significant amounts of angiopoietin-1 (Ang-1) protein when they are engineered into cell sheets cultured for 21 days, including 19 days of osteogenic differentiation [34]. Ang-1 is known to promote endothelial cell survival/migration and the maintenance of endothelial tubular-like structures [55]. The ECM assembled in our cell sheets is enriched in collagen type I presenting a topology favorable to endothelial cell attachment and tubulogenesis [33,34]. A study of Chen et al. also reported that osteogenic cell sheets can promote capillary network formation and development through a higher content of matrix proteins, such as collagen type I and fibronectin [56]. Their investigation of the impact of endothelial cell densities indicated an optimal HUVEC seeding density of 5 × 10^4^ cells cm^−2^ under their experimental conditions [56], which is five times higher than for our method. We believe our lower seeding density allowed the formation of the more extended network of individual capillaries. Taken together, these results suggest that the composition of the ECM, the type of cells that are co-cultured, the seeding density of the endothelial cells, as well as the time allowed for network formation collectively contribute to the quality of the resulting capillary-like networks in engineered tissues.

In the current study, a mineralized endogenous ECM was formed over the 35 days of culture leading to the production of prevascularized and non-prevascularized bone-like tissues. Indeed, hASCs can simultaneously undergo osteogenic differentiation [57] and secrete endogenous ECM when cultured in the presence of osteogenic inducers and ascorbic acid [33,34]. While similar calcium deposition was quantified in the ECM of our current prevascularized bone-like tissue constructs derived from hASCs compared to non-prevascularized tissues produced and described in our previous work [33], the in vitro prevascularization process reduced osteogenesis and hydroxyapatite formation. This is in agreement with Kuss et al., showing that the coculture of ASCs with HUVECs, in both 2D culture and 3D encapsulation systems, does not significantly improve osteogenic differentiation in vitro [58].

Our results also show that levels of osteocalcin deposited in vitro within the endogenous ECM was significantly reduced at 35 days of culture in presence of endothelial cells. Osteocalcin has an important role in bone formation. This non-collagenous bone matrix protein is preferentially expressed by osteoblasts under the control of the RUNX2/CBFA1 transcription factor [59]. Endothelial cells can secrete BMPs [15,52,60], which can upregulate the RUNX2/CBFA1 transcription factor via the SMAD signaling pathway [61], and consequently increase the secretion of osteocalcin surrounding osteogenic cells. However, in a study by Meury et al., hBMSCs were cocultured with HUVECs for 28 h using an indirect culture system (inserts), in the presence or absence of conditioned media enriched in HUVEC-secreted VEGF [62]. Their results showed that HUVECs can inhibit alkaline phosphatase activity, *osterix* (*OSX*) gene expression, and matrix mineralization of hBMSC-derived cell sheets [62]. The OSX transcription factor plays a role in the terminal differentiation of osteoblast cells and the lineage specification between osteogenic and chondrogenic pathways [63]. Even if the present study was performed on a different cell type, their results may explain the reduction of osteocalcin levels we observed in presence of HUVECs.

Our work investigated the capacity of the prevascularized bone-like substitutes to contribute to the in vivo repair of calvarial bone defects created in immunodeficient rats. Surprisingly, the grafted bone-like tissue constructs comprising HUVECs did not result in increased bone formation in vivo when compared to other conditions. No statistically significant differences were seen between the experimental groups following micro-CT imaging and analysis after 12 weeks of implantation. Similarly, Roux et al. recently showed that co-cultured hBMSCs/human induced pluripotent stem cell (iPSC)-derived endothelial cell spheroids seeded within hydroxyapatite/fibrin composite scaffold accelerate vascularization but not bone regeneration in calvarial bone defects of athymic RNU nude rat model after eight weeks post-implantation [64].

Interestingly, the initial difference (1.6-fold) in levels of hydroxyapatite we measured in vitro between the prevascularized and non-prevascularized bone-like tissues did not significantly impact new bone formation in vivo at the implantation sites after 12 weeks. Our findings are consistent with those of Sahar et al. that reported comparable results after grafting, within calvarial bone defects, of a poly(D,L-lactide) acid (PLA) scaffold seeded with rat ASCs and endothelial progenitor cells isolated from the stromal vascular fraction [65]. In their study, experimental groups were composed of acellular PLA scaffold (control), undifferentiated rat ASCs, osteogenically differentiated ASCs (ASC-Osteo), or endothelial differentiated ASCs (ASC-Endo) evaluated eight weeks after implantation using micro-CT analysis and histologic staining [65]. Their results show that the prevascularization of PLA scaffolds with ASC-Endo cells does not increase in vivo bone formation in their experimental setting and the authors suggested that the signals from the endothelial cells may limit the recruitment and the in vivo differentiation of the bone-forming cells of the host.

Despite the non-significant improvement of mature bone formation at the time-point evaluated, our results suggest that the in vitro prevascularization of the bone-like substitutes can improve the survival of the grafted tissues compared to other substitutes after 12 weeks of implantation. However, we acknowledge limitations associated with our preliminary in vivo study. First, although the same protocols were used, in vitro and in vivo experiments were performed independently. Thus, it remains difficult to directly correlate in vitro observations concerning the extent of network development and mineralization to the in vivo results. Second, the in vivo experiments should be repeated with a higher number of grafts and additional time-points to confirm the outcomes. In addition, nude rats were used to evaluate bone healing in this study. However, the immunodeficient status of this animal model does not allow the generation of a normal inflammatory response at the implantation sites, which has been shown to play an important role in bone repair, depending on its intensity [66]. This may explain in part the limited amount of new bone formation measured by micro-CT.

The mechanisms and pathways activated by the interactions between osteoprogenitors and endothelial cells in engineered tissues are still not fully understood and are highly dependent of the specifics of the culture conditions used. Hence, the prevascularized bone-like tissue model we present has the potential to be used as a screening platform to assess how osteogenic genes, such as *RUNX2* or *OSX*, are impacted during the in vitro co-culture of osteogenically induced hASCs with endothelial cells. Finally, in order to further enhance in vitro osteogenesis and mineralization, the use of recombinant BMPs from the transforming growth factor-ß superfamily could be considered since their osteogenic properties are well established in vitro, as well as in vivo, in particular for studies assessing ectopic bone formation [67,68]. For example, the addition of recombinant BMP-2 or BMP-6 to the osteogenic medium may considerably improve in vitro osteogenesis of mesenchymal stem cells and in vivo bone regeneration [69,70,71,72,73]. Under our specific culture conditions, the association of BMPs with a prevascularization strategy may lead to the production of human bone-like substitutes with enhanced osteogenic properties, translating into improved bone repair in vivo.

## 5. Conclusions

Finally, we developed human bone-like tissue substitutes that can be easily prevascularized in vitro using the self-assembly method of tissue engineering. These natural biomimetic constructs supported the formation of well-organized and dense capillary networks in vitro. However, this was associated with reduced osteogenesis and biomineralization in vitro. Once grafted, the prevascularized substitutes supported cell survival in the grafts without impeding in vivo bone healing 12 weeks after implantation. Overall, this model represents a valuable in vitro biological platform to investigate bone physio-pathologies or bone-associated angiogenic processes mediated by various angiomodulators.

## Figures and Tables

**Table 1:** Media composition over time in culture for the in vitro production of the substitutes.

	D3 to D10	D10 to D21	D21 to D35
Osteogenic medium for bone-like substitutes + HUVEC and − HUVEC (final concentration)	Dulbecco’s modified Eagle’s medium (DMEM)	DMEM	DMEM
	Fetal calf serum (FCS) (10%)	FCS (10%)	FCS (5%)
	Penicillin (100 U mL^−1^)	Penicillin (100 U mL^−1^)	Penicillin (100 U mL^−1^)
	Gentamicin (25 μg mL^−1^)	Gentamicin (25 μg mL^−1^)	Gentamicin (25 μg mL^−1^)
	Calcium chloride (1.8 × 10^−3^ M)	Calcium chloride (1.8 × 10^−3^ M)	Calcium chloride (1.8 × 10^−3^ M)
	Sodium L-ascorbic acid (50 μg mL^−1^)	Sodium L-ascorbic acid (50 μg mL^−1^)	Sodium L-ascorbic acid (50 μg mL^−1^)
	Dexamethasone (10 × 10^−9^ M)	Dexamethasone (10 × 10^−9^ M)	Dexamethasone (10 × 10^−9^ M)
	1α,25-dihydroxyvitamin D3 (10 × 10^−9^ M)	1α,25-dihydroxyvitamin D3 (10 × 10^−9^ M)	1α,25-dihydroxyvitamin D3 (10 × 10^−9^ M)
	Ascorbate-2-phosphate (50 × 10^−6^ M)	Ascorbate-2-phosphate (50 × 10^−6^ M)	Ascorbate-2-phosphate (50 × 10^−6^ M)
		ß-glycerophosphate (3.5 × 10^−3^ M)	ß-glycerophosphate (3.5 × 10^−3^ M)
			Endothelial cell growth basal medium-2 (EBM-2^TM^; CC-3156)
			EGM^TM^-2 MV SingleQuots^TM^ Supplement Pack (CC-4147): Fetal bovine serum (FBS) (2.5%) Hydrocortisone Human basic FGF Vascular endothelial growth factor (VEGF) R3-IGF-1 Human EGF
Basal medium for stromal substitutes + HUVEC and − HUVEC (final concentration)	DMEM	DMEM	DMEM
	FCS (10%)	FCS (10%)	FCS (5%)
	Penicillin (100 U mL^−1^)	Penicillin (100 U mL^−1^)	Penicillin (100 U mL^−1^)
	Gentamicin (25 μg mL^−1^)	Gentamicin (25 μg mL^−1^)	Gentamicin (25 μg mL^−1^)
	Calcium chloride (1.8 × 10^−3^ M)	Calcium chloride (1.8 × 10^−3^ M)	Calcium chloride (1.8 × 10^−3^ M)
	Sodium L-ascorbic acid (50 μg mL^−1^)	Sodium L-ascorbic acid (50 μg mL^−1^)	Sodium L-ascorbic acid (50 μg mL^−1^)
			EBM-2^TM^
			EGM^TM^-2 MV SingleQuots^TM^ Supplement Pack

## Data Availability

The data presented in this study are available on request from the corresponding author.

## Supplementary Materials

The following are available online at https://www.mdpi.com/article/10.3390/ma14082023/s1, Figure S1: Thickness of the final constructs after 35 days of culture, Figure S2: Immunodetection of CD31 in bone-like and stromal tissues not supplemented with HUVECs (negative controls).
