# Supplementary material for: In Vitro Prevascularization of Self-Assembled Human Bone-Like Tissues and Preclinical Assessment Using a Rat Calvarial Bone Defect Model
Source: Materials (Basel). 2021 Apr 17;14(8):2023. doi: 10.3390/ma14082023 (PMC8073395; doi:10.3390/ma14082023)
Supplement: Supplementary file 1 [file materials-14-02023-s001.zip › materials-1181322-supplementary.pdf]

materials

Supplementary Materials

In Vitro Prevascularization of Self-Assembled Human Bone-Like Tissues and Preclinical Assessment Using a Rat Calvarial Bone Defect Model

Fabien Kawecki [1,2,†], Todd Galbraith [1,†], William P. Clafshenkel [1], Michel Fortin [1,3,4], François A. Auger [1,2] and Julie Fradette [1,2,*]

1 Centre de Recherche en Organogénèse Expérimentale de l'Université Laval/LOEX, Division of Regenerative Medicine, CHU de Québec Research Center-Université Laval, Québec, QC G1J 1Z4, Canada; fabien.kawecki.1@ulaval.ca (F.K.); Todd.Galbraith@crchudequebec.ulaval.ca (T.G.); bill.clafshenkel@gmail.com (W.P.C.); Michel.Fortin@fmd.ulaval.ca (M.F.); Francois.Auger@fmed.ulaval.ca (F.A.A.)
2 Department of Surgery, Faculty of Medicine, Université Laval, Québec, QC G1V 0A6, Canada
3 Faculty of Dentistry, Université Laval, Québec, QC G1V 0A6, Canada
4 Service of Oral and Maxillofacial Surgery, CHU de Québec-Université Laval, Québec, QC, G1J 1Z4, Canada
* Correspondence: Julie.Fradette@chg.ulaval.ca
† These authors contributed equally to this work.

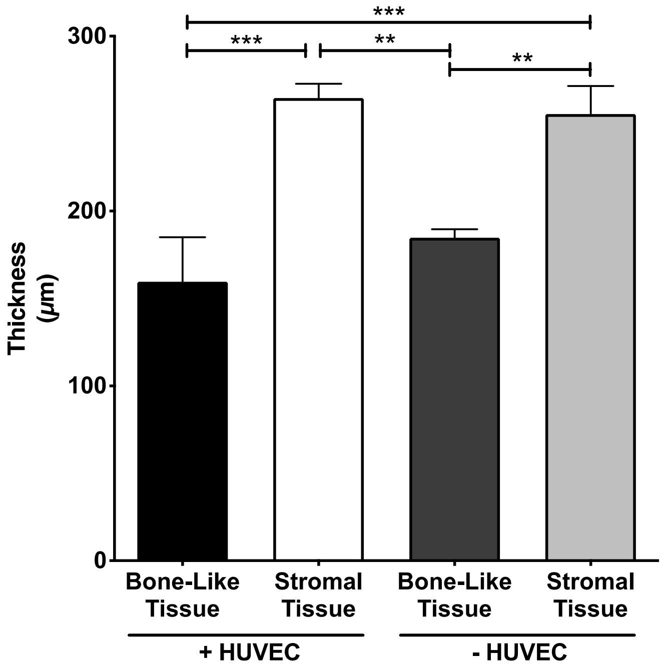

Figure S1. Thickness of the final constructs after 35 days of culture. Measurements on fresh unfixed and unprocessed tissues were obtained using a Biomomentum Mach-1 touch contact option (Mach-1 Biomomentum mechanical testing device, Laval, QC, Canada). Briefly, a flat compression head came in contact with the platform to set origin. Then, tissues were placed on the platform, and the head came down until contact to define its thickness. Prevascularization did not modify the thickness of the bone-like and stromal tissues. However, tissue thickness was significantly reduced after osteogenic induction. One-way ANOVA with a Bonferroni's multiple comparisons test, ** $P < 0.01$, *** $P < 0.001$ ($n = 3$).

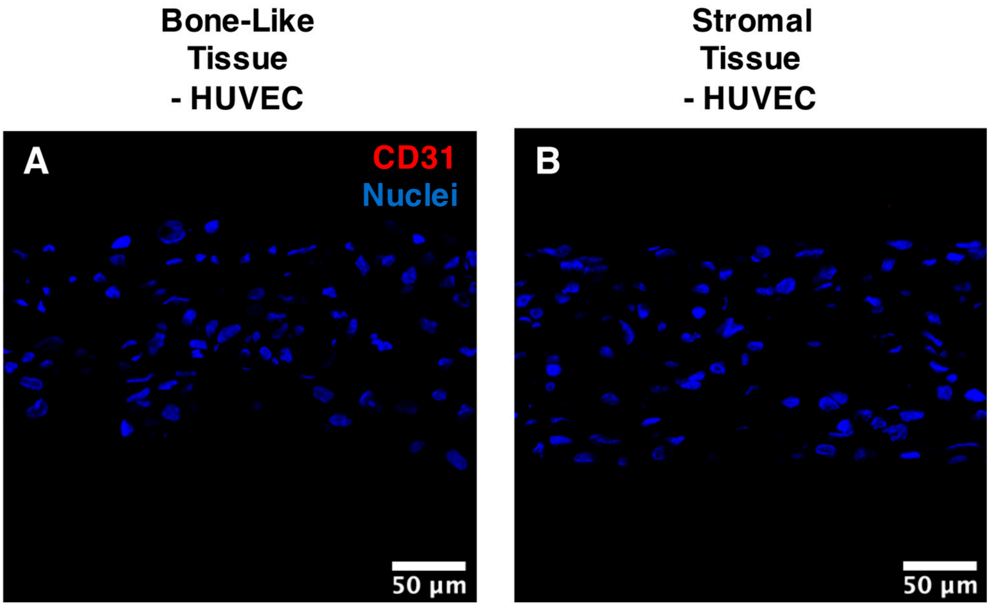

Figure S2. Immunodetection of CD31 in bone-like and stromal tissues not supplemented with HUVECs (negative controls). The absence of CD31-positive structures indicates that the hASC populations used do not contain a subpopulation of cells with the capacity to express CD31 and form capillary networks in the absence of HUVECs in bone-like (A) and stromal (B) tissues under these culture conditions. Nuclei were stained in parallel with Hoechst (blue). Scale bars, 50 µm.
